# Yolk–shell structured magnetic mesoporous silica: a novel and highly efficient adsorbent for removal of methylene blue

**DOI:** 10.1038/s41598-021-02699-w

**Published:** 2021-12-01

**Authors:** Reza Mirbagheri, Dawood Elhamifar, Masoumeh Shaker

**Affiliations:** Department of Chemistry, Yasouj University, 75918-74831 Yasouj, Iran

**Keywords:** Analytical chemistry, Environmental chemistry, Green chemistry, Process chemistry

## Abstract

In this study, a novel magnetic mesoporous silica with yolk–shell structure (Fe_3_O_4_@Void@m.SiO_2_) was successfully synthesized via a polymer-template assisted method. The Fe_3_O_4_@Void@m.SiO_2_ was characterized by using FT-IR, EDS, SEM, TEM, VSM, PXRD and nitrogen adsorption–desorption analyses. The Fe_3_O_4_@Void@m.SiO_2_ nanocomposite showed high efficiency in adsorption of an organic dye and water pollutant called methylene blue (MB) with 98.2% removal capability. Furthermore, the effect of different parameters in the adsorption of MB was investigated. Different models of kinetic were examined and compared with each other. The recoverability and reusability of designed Fe_3_O_4_@Void@m.SiO_2_ material were also studied under applied conditions.

## Introduction

After the first report about interesting materials with yolk–shell (YS) structure^1^, many researches have developed the synthesis methods and properties of these materials with different names such as nanorattle^2^, movable core/shell^1^, core/shell with hollow interiors^3^ and yolk/shell^4^. These nanomaterials are in the center of attention due to interesting properties such as high surface area, interstitial hollow space and low density^5,6^. These special properties make yolk–shell nanocomposites suitable to use in the fields of biomedical^7^, lithium batteries^8,9^, sensors^10,11^, catalysis^12–16^ and adsorption^17,18^. Some of recently reported yolk–shell structured materials are Sn_4_P_3_@C^19^, Sn@SnO/SnO_2_^20^, Al@TiO_2_ NPs^21^ and Au-CeO_2_@ZrO_2_^22^. Among different shells, silica-based ones is more important in catalysis and adsorption processes owing to its high loading capacity compared to other shells^23,24^. The preparation methods effect on features of the yolk, space, thickness, porosity and shape of YS materials. The more common methods used for the preparation of YSs are hard temple-assisted, soft template-assisted and template-free^25^. Among these, the soft template method has attracted more attention due to easy removal of template and also economically friendly^26,27^.

Moreover, owing to superparamagnetic properties, easy separation and low toxicity, magnetic nanoparticles (MNPs) have been so interested in different fields such as biomedical^28,29^, magnetic resonance imaging^30^, drug delivery^31^ and separation^32^. However, the most of MNPs suffer from disadvantages of aggregation, biodegradation and low capacity. In order to increase the stability and capacity, these NPs are composited with different species. Among different species, mesoporous silica is more attracted because of its high surface area, high pore volume and high capacity^33–35^.

As regards to water purification importance and lots of different pollutants that made by pharmaceutical, paper making, textile, leather, etc., different methods such as photocatalytic degradation, adsorption and oxidation have been used to eliminate water pollutants^36,37^. To date many adsorbents have been applied for the elimination of organic and inorganic pollutants from water. Among these, magnetic NPs have attracted more attention due to the advantages of easy magnetically separation, economically friendly and high efficiency. Some of recently developed systems are magnetic zeolites^38^, magnetic carbon nanotubes composites^39^, Zn/ferrite/graphene oxide^40^ and activated carbon/NiFe_2_O_4_^41^. Although these adsorbents gave good to high efficiency in the removal of water pollutants, however, in spite of high adsorption capacity of the yolk–shell based magnetic nanocomposites, according to our knowledge, there is no report in the study of efficiency of these type nanomaterials in the removal of water pollutants. In view of the above, herein, a novel magnetic Fe_3_O_4_@mesoporous silica nanocomposite (Fe_3_O_4_@Void@m.SiO_2_) with yolk–shell structure and high magnetic properties is prepared for adsorption of methylene blue (MB) from water. The kinetics, isotherm and equilibrium data for adsorption of MB have been analyzed and different models have been employed to understand the mechanism.

## Experimental section

### Preparation of Fe_3_O_4_@Void@m.SiO_2_ nanomaterial

**Figure 1:**
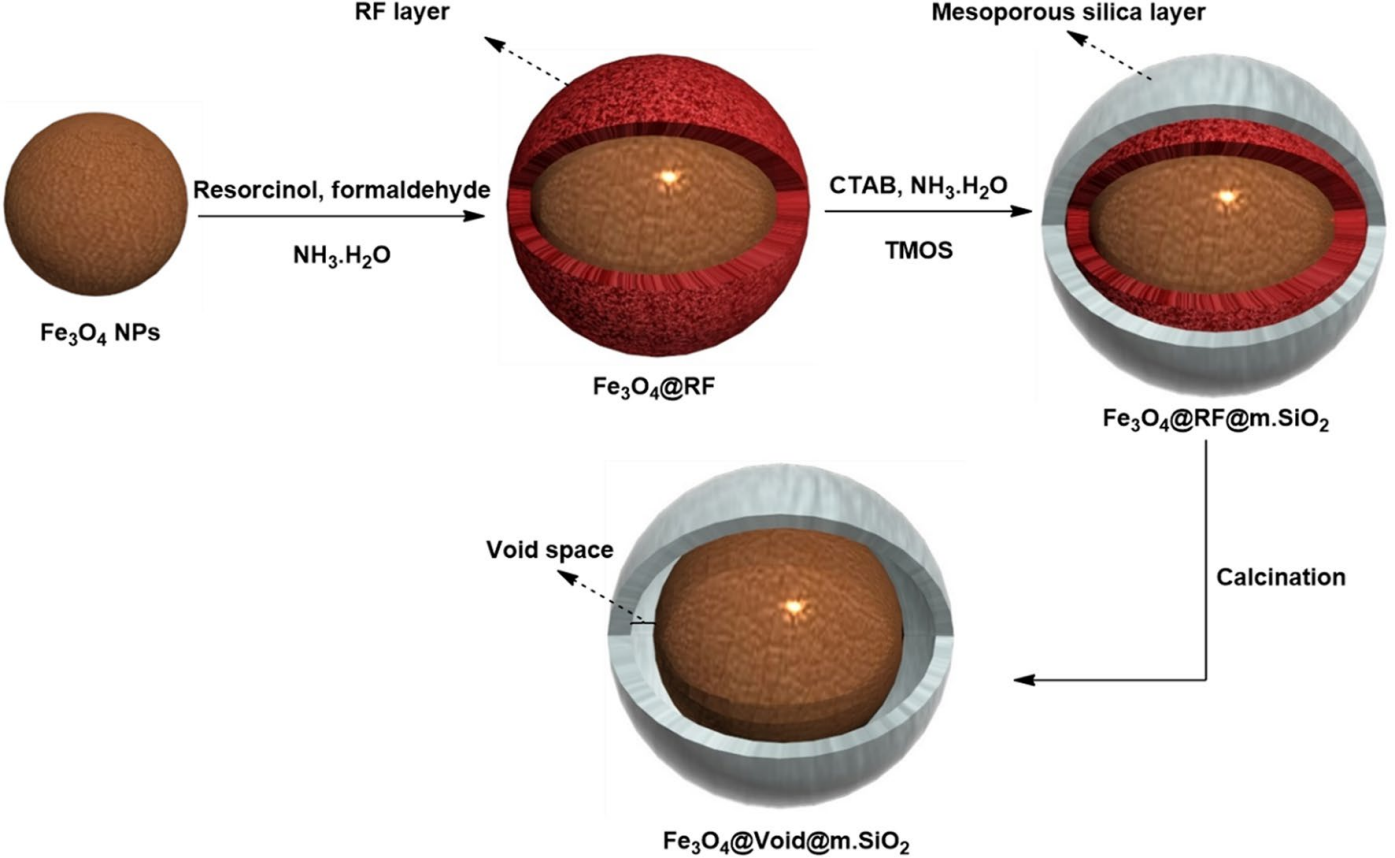
Preparation of Fe_3_O_4_@Void@m.SiO_2_.

To do this, firstly magnetic Fe_3_O_4_ NPs were prepared^42^. In the second step, the Fe_3_O_4_ NPs were modified with resorcinol–formaldehyde polymer to form Fe_3_O_4_@RF material. In order to prepare Fe_3_O_4_@RF, the Fe_3_O_4_ NPs (100 mg) were added to a mixture of EtOH (20 mL) and H_2_O (10 mL) in an ultrasonic bath. Then, ammonium hydroxide (0.5 g, aqueous solution, 28 wt %), HCHO (0.1 g, 37 wt %) and resorcinol (0.1 g, 0.09 mM) were added while stirring for 2 h. After polymerization, the resulted Fe_3_O_4_@RF was collected and washed completely with H_2_O and EtOH. Next, the as-made Fe_3_O_4_@RF (0.12 mg) was dispersed in a mixture of CTAB (0.45 g), ammonia (2 mL, 28 wt %), H_2_O (100 mL) and EtOH (150 mL). The resulted combination was stirred to form a homogeneous mixture. After adding of TMOS (1.5 mL), stirring was continued for 6 h. The resulted Fe_3_O_4_@RF@m.SiO_2_ material was washed completely with EtOH and H_2_O. Finally, the RF layer and CTAB surfactant were removed after heating of the Fe_3_O_4_@RF@m.SiO_2_ material at 550 °C for 6 h (Fig. 1). The final magnetic nanomaterial with yolk–shell structure was denoted as Fe_3_O_4_@Void@m.SiO_2_.

### Adsorption process

For this, 5 mg of the Fe_3_O_4_@Void@m.SiO_2_ was added in 15 mL of an aqueous solution of MB. This mixture was shaken continuously and the Fe_3_O_4_@Void@m.SiO_2_ was magnetically separated immediately. The MB concentration was measured by UV–vis at 665 nm. The adsorption efficiency for MB in water solution was calculated by using Eq. (1).

1 $${\text{adsorption efficiency }}\left( {\text{\% }} \right) = \frac{{C_{0} - C_{e} }}{{C_{0} }} \times 100$$

On the other hand, the amount of adsorbed MB on the adsorbent (q_e_) was calculated by using Eq. (2).

2 $${\text{q}}_{e} = \frac{{(C_{0} - C_{e} ) \times V}}{m}$$

Herein, C_0_ and C_e_ are, respectively, the concentration of the initial dye solution and the residual dye solution that quantitatively estimated by linear regression equations resulted at different dye concentrations, V is solution volume in liters and m is adsorbent amount (mg).

## Results and discussion

**Figure 2:**
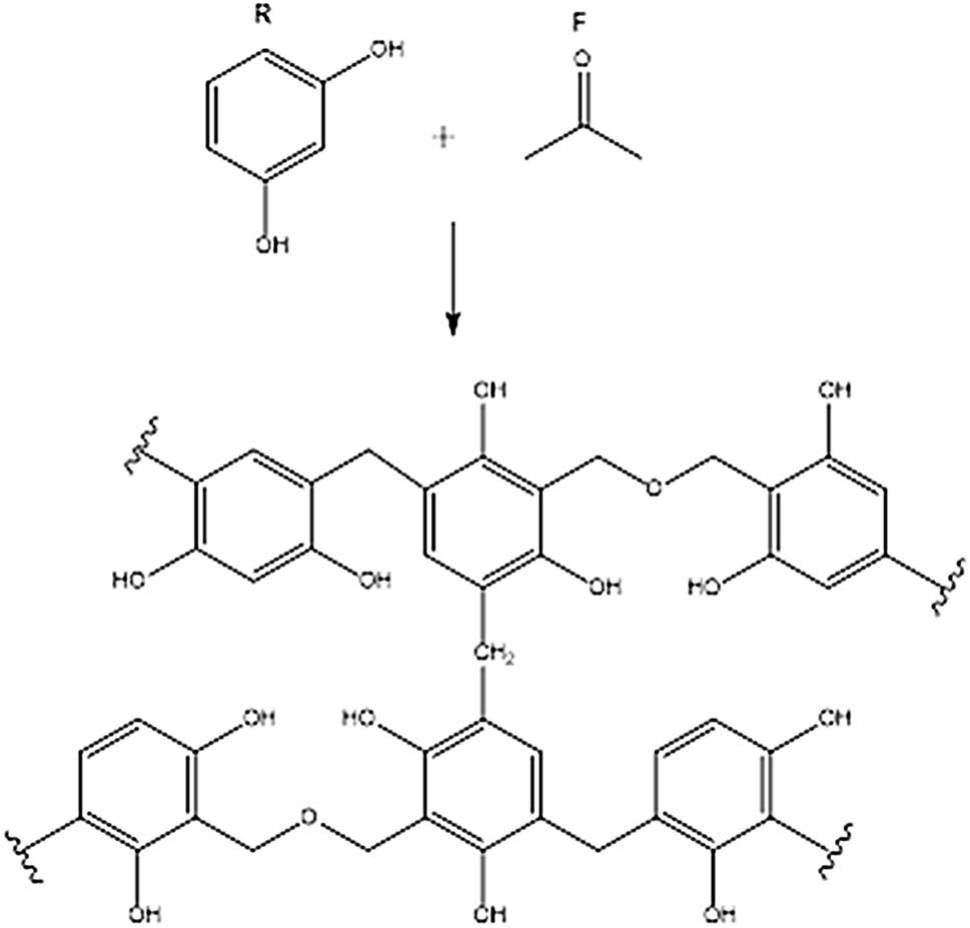
A suitable pathway for polymerization of formaldehyde and resorcinol.

The magnetic mesoporous silica material (Fe_3_O_4_@Void@m.SiO_2_) with yolk–shell structure was prepared from an intermediate structure (Fe_3_O_4_@RF) which was made by polymerization of HCHO and resorcinol (Fig. 2) on the Fe_3_O_4_NPs surface. The mesoporous silica shell was coated on the Fe_3_O_4_@RF by using CTAB and TMOS via a sol–gel approach. Finally, RF and CTAB were eliminated to deliver Fe_3_O_4_@Void@m.SiO_2_ (Fig. 1).

### Characterization

**Figure 3:**
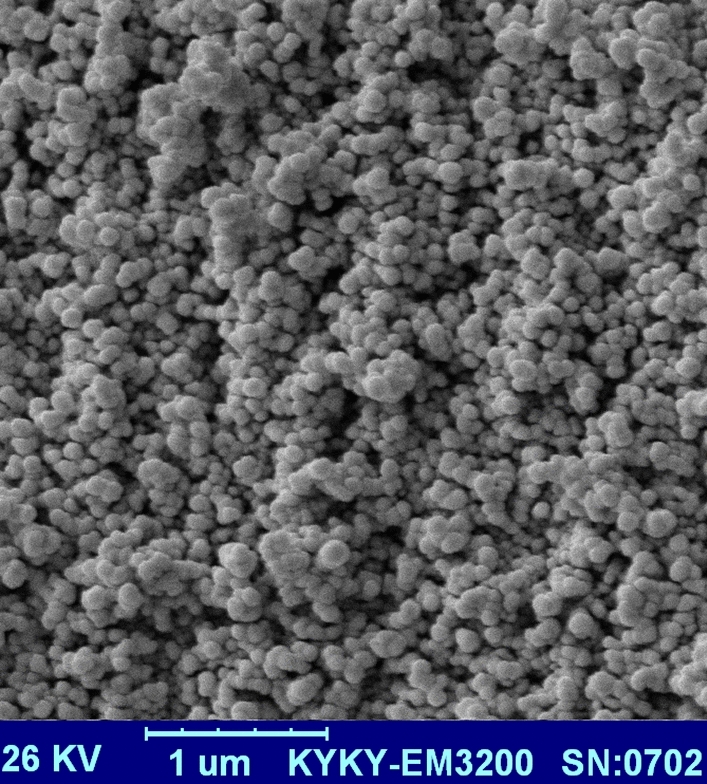
SEM image of the Fe_3_O_4_@Void@m.SiO_2_ nanomaterial.

The SEM image of Fe_3_O_4_@Void@m.SiO_2_ yolk shell nanomaterial is illustrated in Fig. 3. As shown, the designed material has particles with spherical morphology and average size of 84 nm. These types of NPs are excellent candidates for adsorption processes.

**Figure 4:**
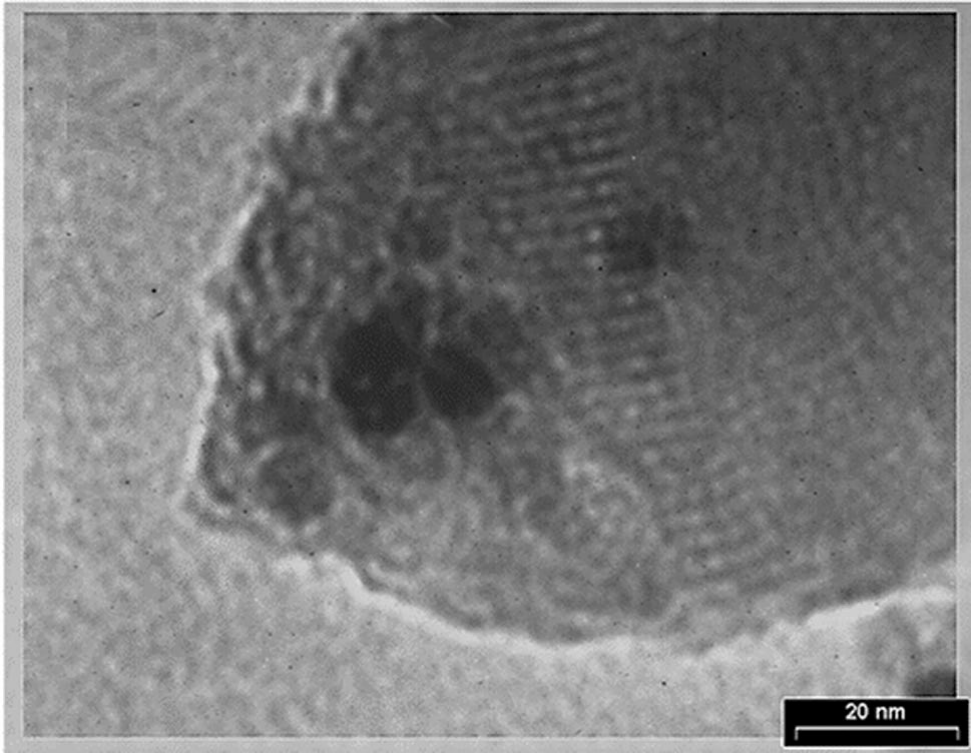
TEM image of Fe_3_O_4_@Void@m.SiO_2_.

The TEM image of the Fe_3_O_4_@Void@m.SiO_2_ nanomaterial also showed a yolk–shell structure with black cores (Fe_3_O_4_ NPs) and mesoporous silica shell (Fig. 4).

**Figure 5:**
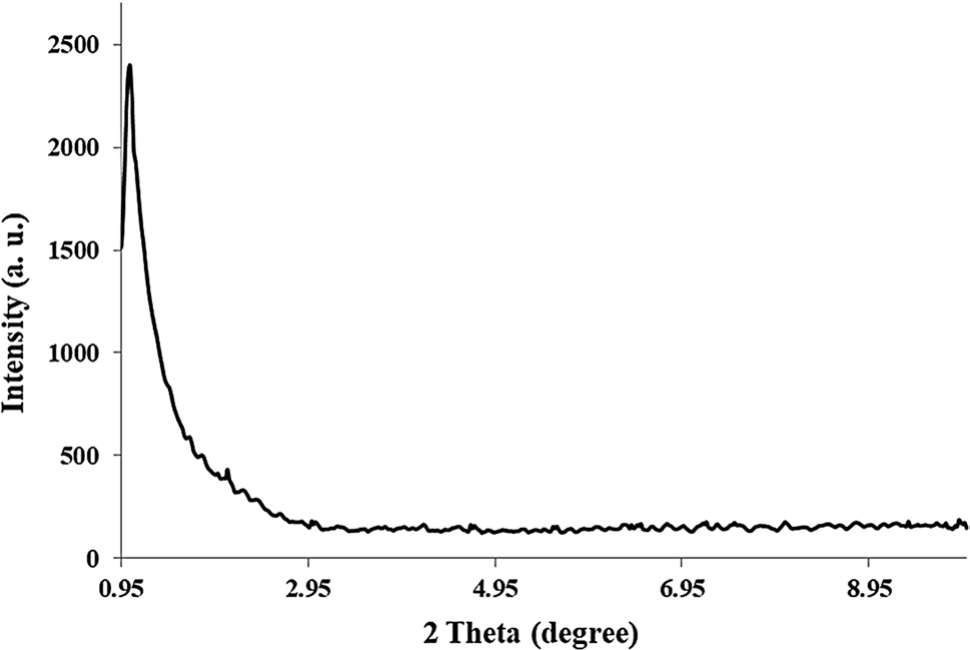
The low angle PXRD pattern of Fe_3_O_4_@Void@m.SiO_2_.

The low-angle PXRD (LA-PXRD) pattern of Fe_3_O_4_@Void@m.SiO_2_ showed an intense peak centered at 2 theta of 1 degree that is characteristic of nanomaterials with an ordered 2D hexagonal mesostructure (Fig. 5). These types of materials have high surface area and are very effective in the adsorption processes.

**Figure 6:**
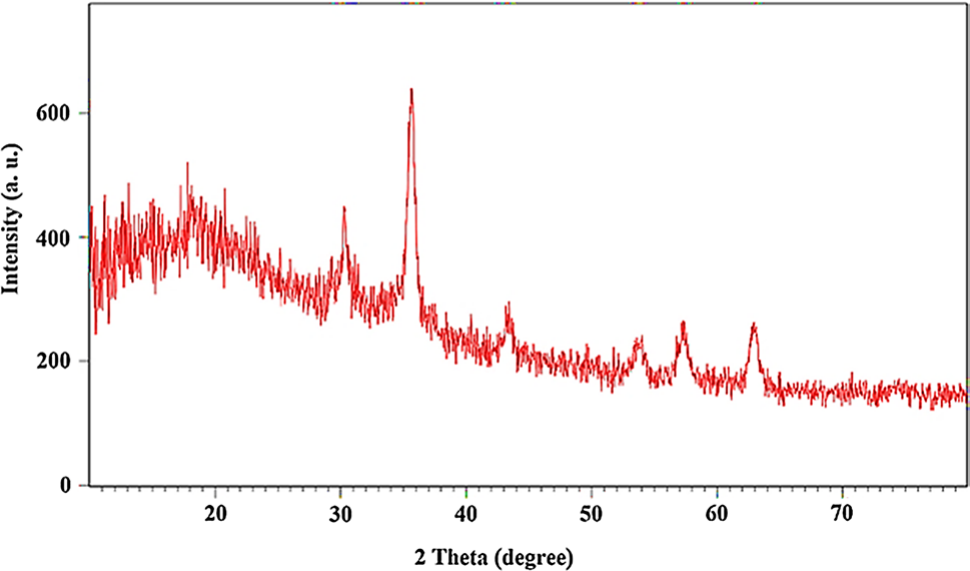
The wide-angle PXRD pattern of Fe_3_O_4_@Void@m.SiO_2_.

The wide-angle PXRD of Fe_3_O_4_@Void@m.SiO_2_ showed six reflection peaks at 2θ of 63°, 57°, 54°, 43°, 35° and 30° (Fig. 6). This pattern is in good agreement with the PXRD pattern of Fe_3_O_4_ NPs confirming high stability of the magnetite cores during material preparation. This analysis also showed a broad peak at 2 theta about 20 degree that is attributed to mesoporous silica shell^42^.

**Figure 7:**
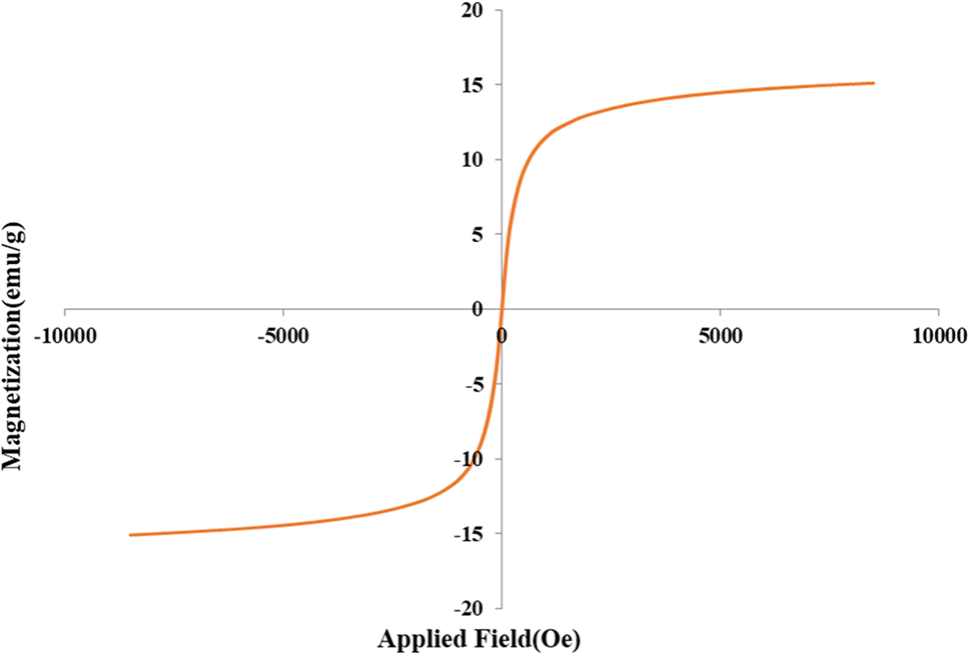
The VSM analysis of Fe_3_O_4_@Void@m.SiO_2_.

Figure 7 shows the vibrating sample magnetometer (VSM) analysis of the designed nanomaterial. As shown, this material has good magnetic property. As expected, due to the nonmagnetic mesoporous silica shell and the large void space, the magnetization is reduced from 60 for Fe_3_O_4_ to 15 emu/g for Fe_3_O_4_@Void@m.SiO_2_^14^.

**Figure 8:**
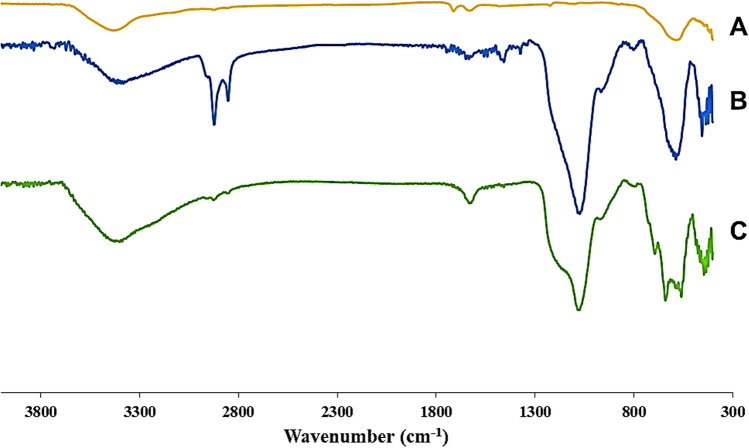
FT-IR results of Fe_3_O_4_ (**A**), Fe_3_O_4_@Void@m.SiO_2_ nanomaterial before (**B**) and after (**C**) calcination.

The FT-IR spectra of Fe_3_O_4_ and Fe_3_O_4_@Void@m.SiO_2_ before and after calcination, are shown in Fig. 8. For all materials, the absorption peaks of Fe–O bonds are observed about 580 cm^−1^. The bands at 710–810 cm^−1^ are due to C–Si. The peaks cleared at 1100 and 935 cm^−1^ are due to Si–O–Si bonds. The peak at 3500 cm^−1^ is owing to O–H bonds of material surface. Before surfactant and RF removal, the peaks at 2850 and 2920 cm^−1^ are corresponded to aliphatic C–H bonds of CTAB and peaks about 3020 cm^-1^ are related to aromatic C–Hs of RF (Fig. 8B). Interestingly, the latter peaks are disappeared in Fig. 8C confirming successful removal of CTAB and RF during calcination. It is important to note that, to prepare a material with mesoporous yolk–shell structure, both CTAB (containing aliphatic C–H) and RF (containing aromatic C–H) should be romed. After removal of CTAB a mesoporous silica shell is resulted, while after removal of RF the void space between shell and core is resulted.

**Figure 9:**
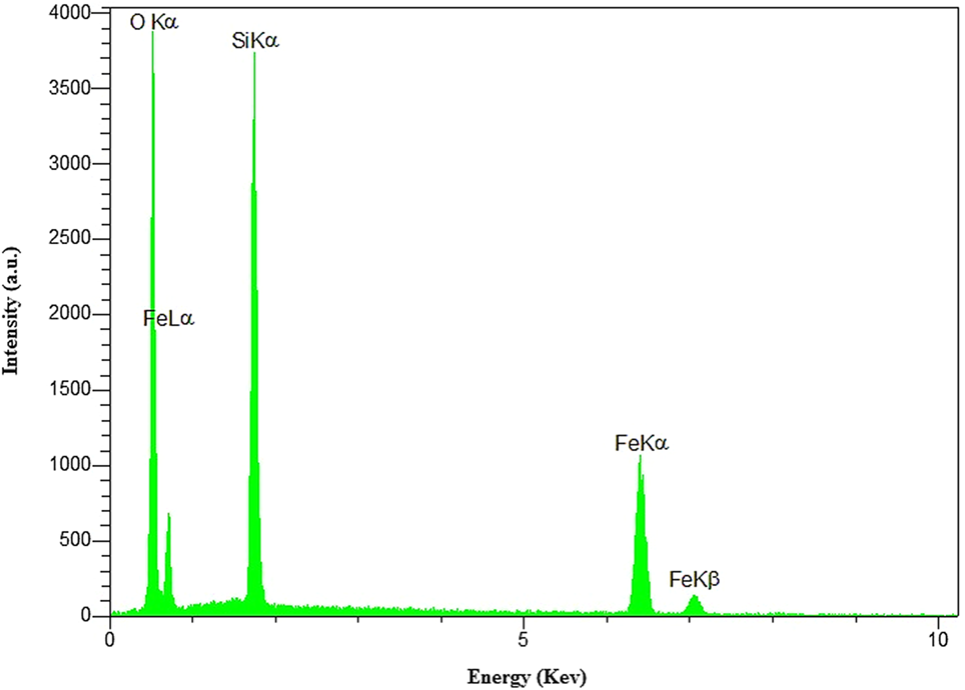
EDS analysis of Fe_3_O_4_@Void@m.SiO_2_ nanomaterial before adsorption process.

The EDS was performed for elemental analysis of the material before adsorption process (Fig. 9). This analysis clearly showed the existence of oxygen, iron and silicon confirming high stability and well incorporation of expected magnetite cores and silica shells in the material network.

**Figure 10:**
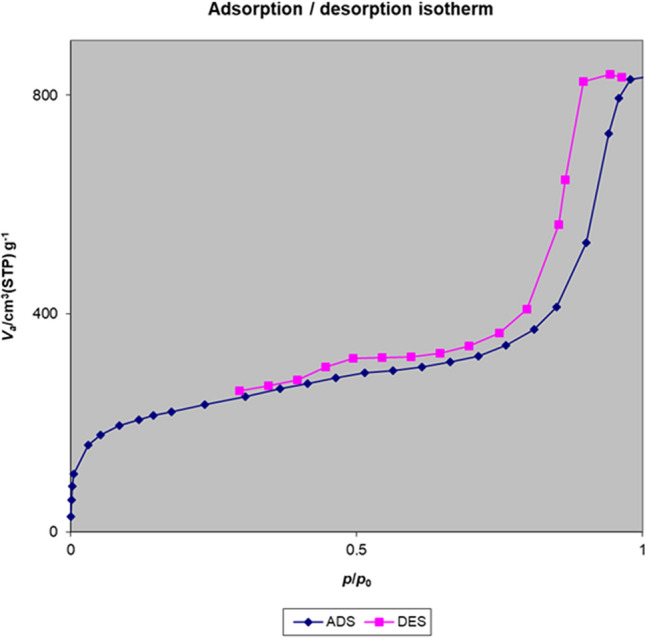
Nitrogen adsorption–desorption isotherm of the Fe_3_O_4_@Void@m.SiO_2_ nanomaterial before adsorption process.

**Figure 11:**
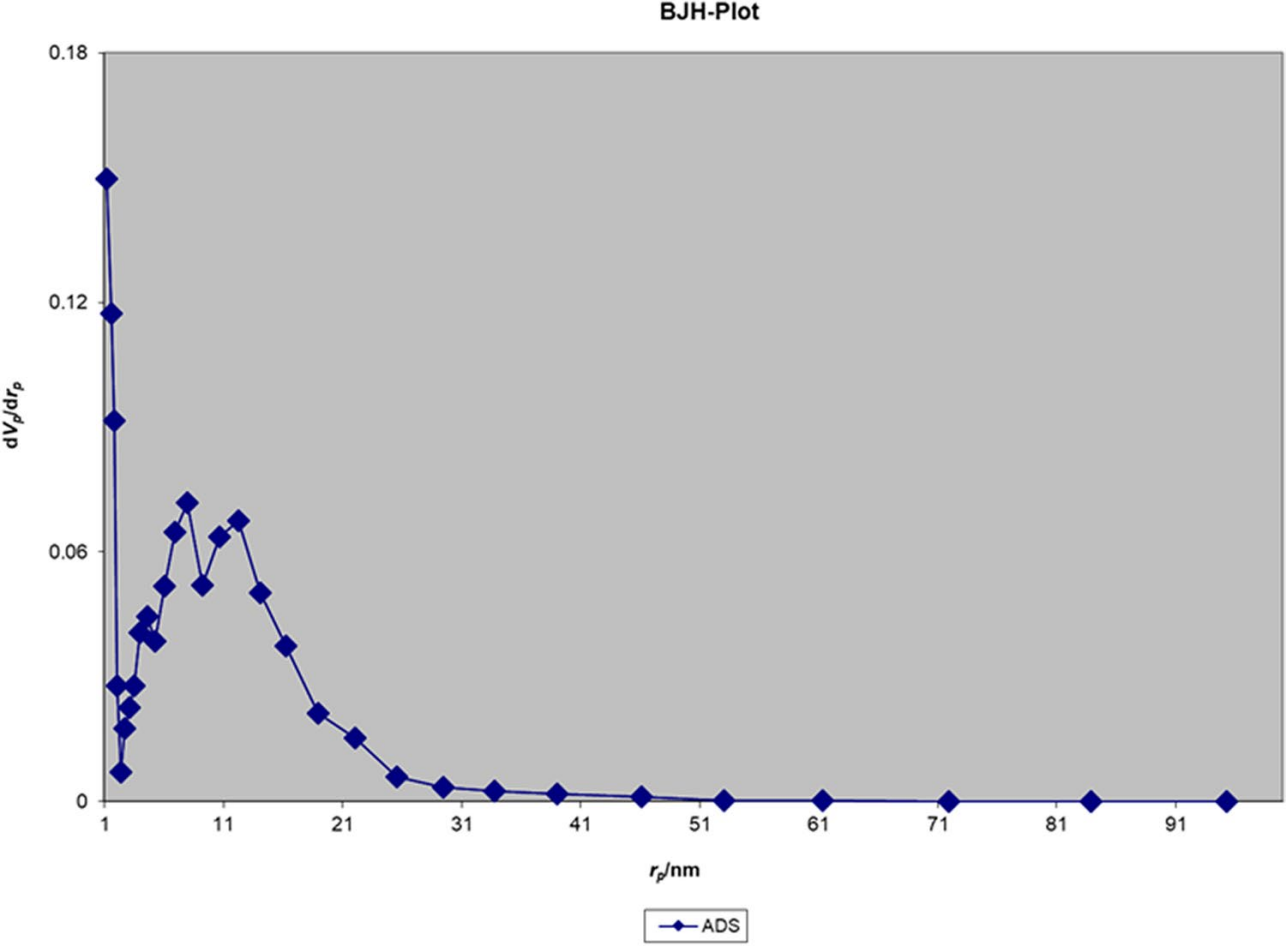
BJH pore size distribution isotherm of the Fe_3_O_4_@Void@m.SiO_2_ nanomaterial before adsorption process.

The nitrogen adsorption–desorption isotherm of the Fe_3_O_4_@Void@m.SiO_2_ nanomaterial before adsorption process shows a type IV isotherm with an H2 hysteresis loop, which is characteristic of ordered mesostructures with high regularity (Fig. 10). The sharp capillary condensation steps occurred at a relative pressure of 0.45–0.97, indicate the large void space of Fe_3_O_4_@Void@m.SiO_2_ and the porous silica shell^43^. The Brunauer–Emmett–Teller (BET) surface area and total pore volume of the material were also found to be 666.16 m^2^/g and 1.28 cm^3^/g, respectively. The Barrett–Joyner–Halenda (BJH) pore size distribution isotherm showed a bimodal size distribution at 7.1 and 12.2 nm related to the mesoporous shell and void space between yolk and shell, respectively (Fig. 11). These data are in good agreement with LA-PXRD and TEM results confirming the presence of a mesoporous shell and yolk–shell structure for the designed nanomaterial.

### Adsorption Studies

The elimination of MB, as organic dye, was examined as a model to study adsorption ability of synthesized Fe_3_O_4_@Void@m.SiO_2_ material. The time, dye concentration, amount of adsorbent and adsorption pH were optimized as well as different models were checked in kinetic and isotherm studies.

#### Effect of pH

**Figure 12:**
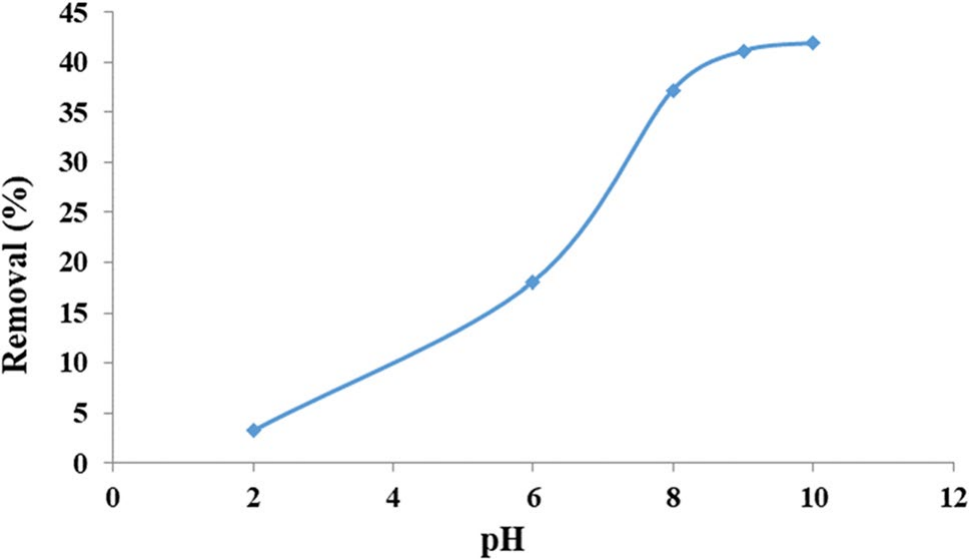
The effect of pH on MB adsorption.

**Figure 13:**
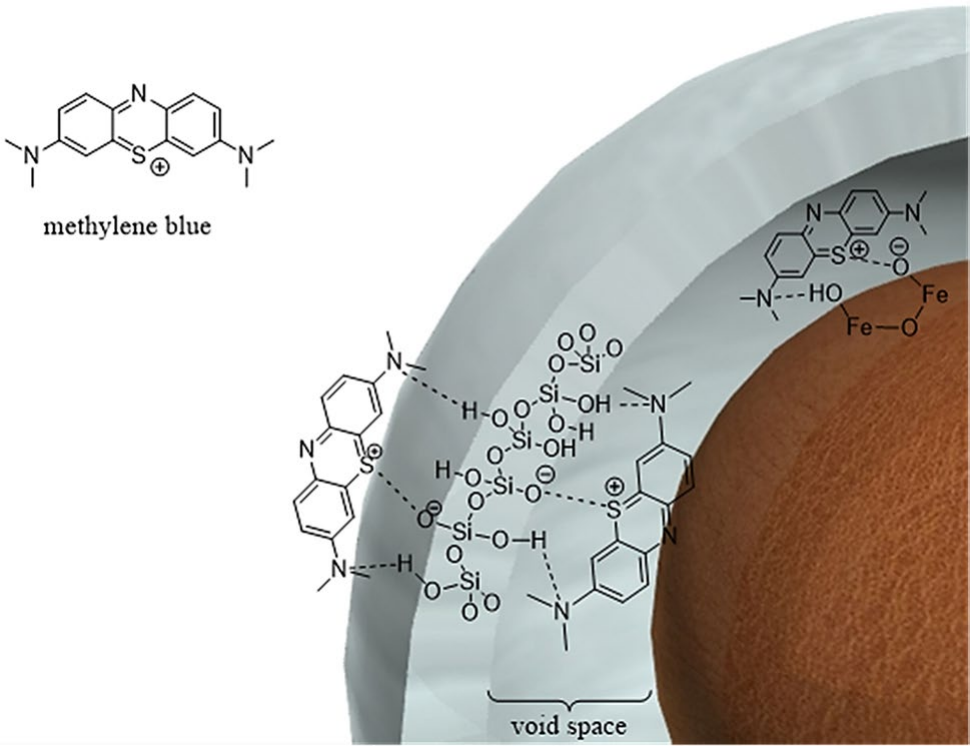
A suitable mechanism for MB adsorption using Fe_3_O_4_@Void@m.SiO_2_.

The effect of pH was investigated due to the structure and ionization degree of the MB dye can be influenced by pH value^44^. The pKa of MB is 3.8^45^, therefore, at pH values above this, the preponderant MBs are cationic. The effect of pH values was studied during 5 min using 0.005 g of the adsorbent (Fig. 12). At acidic pH, the –OHs of mesoporous silica shell and magnetic core interact with MBs via H-bonding. At basic pH, Si–O− and Fe–O− are the greater groups due to the deprotonation of Si–OH and Fe–OH, respectively. These sites improve the electrostatic interaction between the cationic dye and the anionic charged surfaces (Fig. 13). Similar results have been reported for MB elimination using clay^46,47^ and activated coal^48^. Another important factor for this adsorption process is H-bonding interactions between N-sites of dye and OH sites of adsorbent. It is important to note that the aforementioned interactions can occur on both inner and outer surfaces of mesoporous silica shell and also on outer surface of magnetite cores^49,50^. According these findings, a suitable mechanism for this adsorption process is proposed in Fig. 13. According to this experiment, at pH 9.0 the best result was obtained. Therefore, this was used as optimum pH in the subsequent tests.

#### Effect of adsorbent dosage and dye concentration

**Figure 14:**
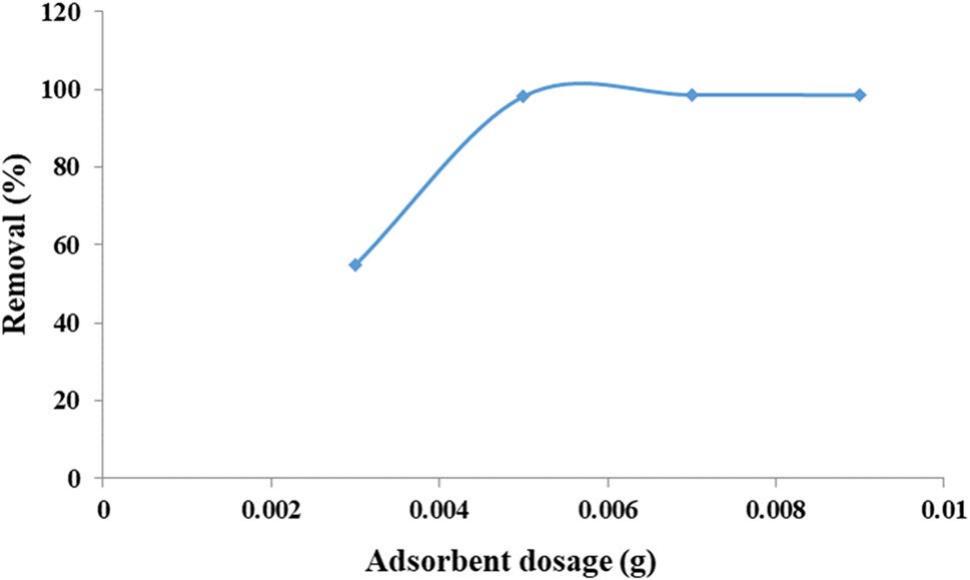
The effect of Fe_3_O_4_@Void@m.SiO_2_ amount on MB adsorption.

**Figure 15:**
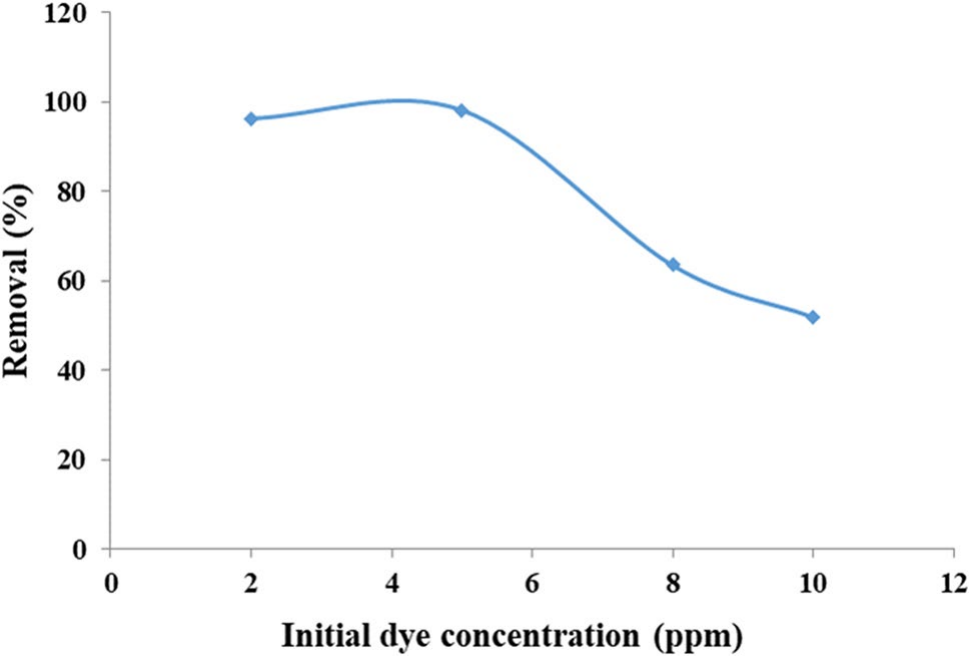
The effect of MB concentration using 0.005 g of adsorbent.

In next step, the amounts of adsorbent and dye concentration were optimized (respectively, Figs. 14 and 15). It is clear that the adsorbent amount and the numbers of active positions on its surface affect the adsorption rate. By increasing the amount of adsorbent, the vacant and unoccupied positions are increased and thus the percentage of removal is increased. While, after this, there is a little change in the adsorption process. As shown in Fig. 14, the optimum amount of adsorbent is 0.005 g for 15 mL of MB aqueous solution (5 ppm) with 98.2% removal. This confirms high performance of the designed material in the MB removal. In the next, the effect of dye concentration using a constant amount (0.005 g) of adsorbent was studied. As shown in Fig. 15, by increasing the amount of MB concentration, the removal performance of a certain amount of Fe_3_O_4_@Void@m.SiO_2_ is reduced which it refers to disproportionate in amount of active sites and dye molecules. Accordingly, for 0.005 g of Fe_3_O_4_@Void@m.SiO_2_ adsorbent, the optimum dye concentration was 5 ppm.

#### Effect of time

**Figure 16:**
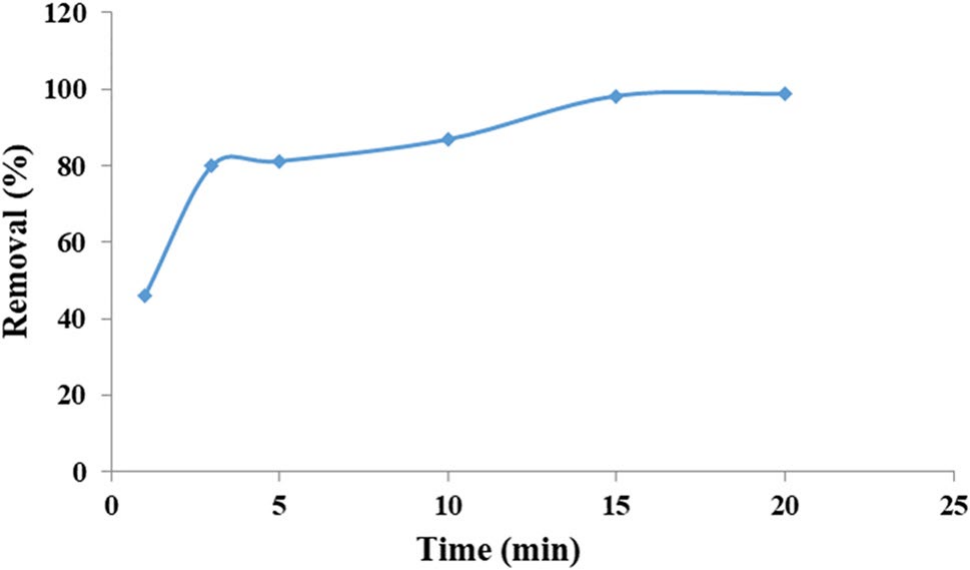
Effect of time.

Figure 16 illustrates the time effect under optimum conditions. As seen, the rate of adsorption is high at first 3 min that can refer to adsorption by the outer surface of the shell. After that, this process is slowly increased with time. The latter adsorption can be happened on internal surface of the shell and also surface of the core. As shown, after 15 min the maximum adsorption is resulted.

#### Kinetics evaluation

**Figure 17:**
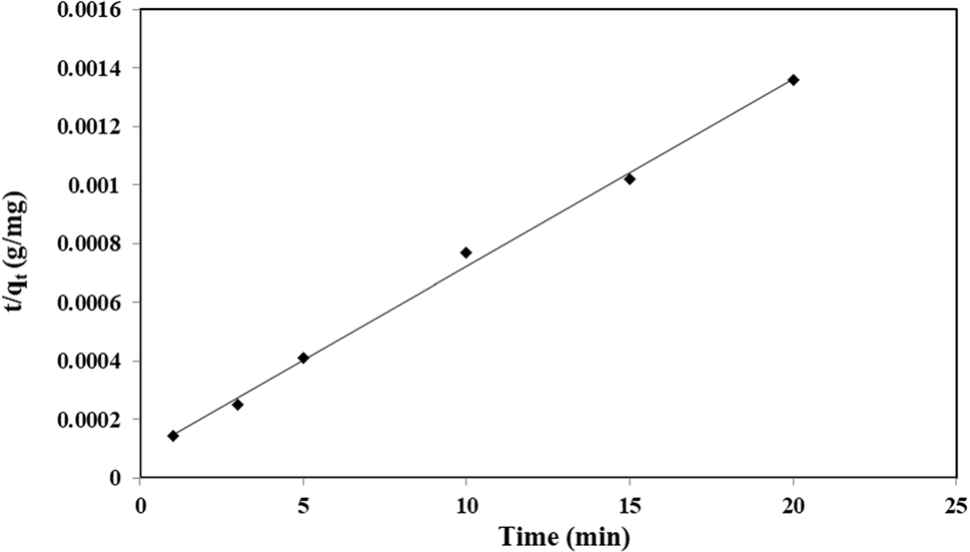
The pseudo-second-order plot of MB adsorption using Fe_3_O_4_@Void@m.SiO_2_.

To study the kinetics evaluation, the kinetic models of pseudo-first-order, second-order and Elovich were employed (Table 1). The pseudo-first-order model is as follows:

3 $$\log (q_{e} - q_{t} ) = \frac{{\log q_{e} - k_{1} t}}{2.303}$$

where q_t_ is the adsorbed dye at t time (mg/g); q_e_ is adsorbed dye at equilibrium (mg/g) and k_1_ is the rate constant of adsorption (min^−1^). The pseudo-second-order model is also as follows:

4 $$\frac{t}{{q_{t} }} = \frac{1}{{k_{2} q_{e}^{2} }} + \frac{t}{{q_{e} }}$$

the q_e_ and k_2_ were obtained via the plot of $\frac{t}{{q_{t} }}$ versus *t* (Fig. 17, Table 1). The Elovich equation is also as follows:

5 $$q_{t} = 1/\beta \ln (\alpha \beta ) + 1/\beta \ln (t)$$

**Table 1:** Kinetic parameters for MB adsorption using Fe_3_O_4_@Void@m.SiO_2_.

Model	Parameters	Adsorbent
		Fe_3_O_4_@Void@m.SiO_2_
First-order kinetic	k_1_	0.093
	qe (calc)	4.959
	R^2^	0.808
Second-order kinetic	k_2_	0.049
	q_e_ (calc)	15.64
	R^2^	0.997
Elovich	Β	0.406
	Α	59.943
	R^2^	0.912

As shown in Table 1 and Fig. 17, the pseudo-second-order model with a linear regression correlation coefficient (R^2^) value of 0.997 is completely applicable for the process.

#### Isotherm study

To determine the maximum capacity of sorption, the Langmuir, Freundlich and Temkin isotherm models were employed. The Langmuir model is as follows:

6 $$q_{e} = q_{m} \frac{{K_{L} C_{e} }}{{1 + K_{L} C_{e} }}$$

where q_e_ is the equilibrium concentration of dye, q_m_ is the maximum dye uptake and K_L_ is Langmuir constant. These are determined by linearizing of Eq. (6) as shown in Eq. (7),

7 $$\frac{{C_{e} }}{{q_{e} }} = \frac{1}{{K_{L} Q_{m} }} + \frac{{C_{e} }}{{Q_{m} }}$$

The Freundlich model is also as follows:

8 $$q_{e} = K_{F} C_{e}^{1/n}$$

The linear form of above equation is Eq. (9) in which K_f_ and n are Freundlich constants

9 $$\log (q_{e} ) = \log (K_{F} ) + \frac{1}{n}\log (C_{e} )$$

The other isotherm model is Temkin with a linear form as follows:

10 $$q_{e} = B_{T} \ln K_{T} + B_{T} \ln C_{e}$$

**Figure 18:**
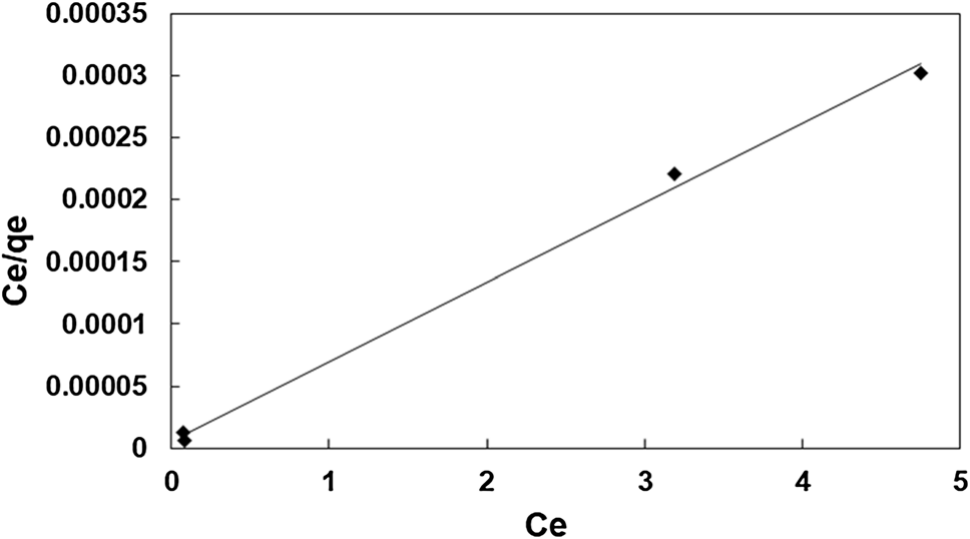
The Langmuir plot of MB adsorption by using Fe_3_O_4_@Void@m.SiO_2_.

As shown in Table 2 and Fig. 18, the Langmuir model with an R^2^ > 0.99 is the best isotherm for the process.

**Table 2:** Isotherm parameters and R^2^ amounts obtained for the MB adsorption using Fe_3_O_4_@Void@m.SiO_2_**.**

Isotherm	Parameters	Adsorbent
		Fe_3_O_4_@Void@m.SiO_2_
Langmuir	Q_m_(mg/g)	163.934
	K_a_ (L/mg)	0.095
	R^2^	0.996
Freundlich	1/n	0.130
	K_F_ (L/mg)	349.945
	R^2^	0.379
Tempkin	B_1_	1.287
	K_T_ (L/mg)	34,166.468
	R^2^	0.393

#### Thermodynamic study

Thermodynamic parameters (ΔG^0^, ΔH^0^ and ΔS^0^) were calculated by using following equations^51^:

11 $$K_{c} = \frac{{q_{e} }}{{C_{e} }}$$

12 $$\Delta G^{0} = - RT\ln K_{c}$$

13 $$\Delta G^{0} = \Delta H^{0} - T\Delta S^{0}$$

14 $$lnK_{c} = \frac{{\Delta S^{0} }}{R} - \frac{{\Delta H^{0} }}{RT}$$

**Figure 19:**
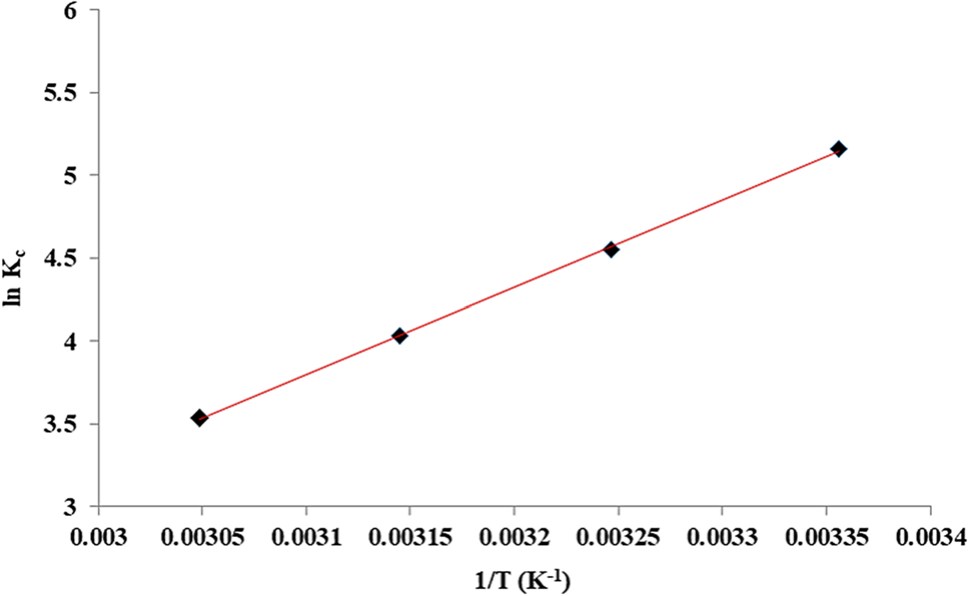
The plot of ln K_c_ versus 1/T.

These parameters are listed in Table 3. The plot of ln K_c_ versus 1/T is illustrated in Fig. 19. As shown, a satisfactory adsorption is resulted at RT and by increasing the temperature from 25 to 55 °C, a slight decrease in MB adsorption is observed indicating the process is exothermic. This was also confirmed by the negative ΔH°. The negative amount of ΔG^0^ confirms that the MB adsorption on Fe_3_O_4_@Void@m.SiO_2_ is achievable and spontaneous. The negative ΔS° also suggests a decrease in randomness at solid/solution interface^52^. Moreover, the amounts of ΔH^0^ and ΔG^0^ successfully indicate that MB molecules are both physically and chemically adsorbed into/onto adsorbent material.

**Table 3:** Thermodynamic parameters of MB adsorption using Fe_3_O_4_@Void@m.SiO_2_.

Temperature (K)	ΔG^0^ (kJ/mol)	ΔH^0^ (kJ/mol)	ΔS^0^ (J/mol K)	R^2^
298	− 12.79	− 43.94	− 104.65	0.999
308	− 11.65			
318	− 10.65			
328	− 9.64

#### EDS spectrum

**Figure 20:**
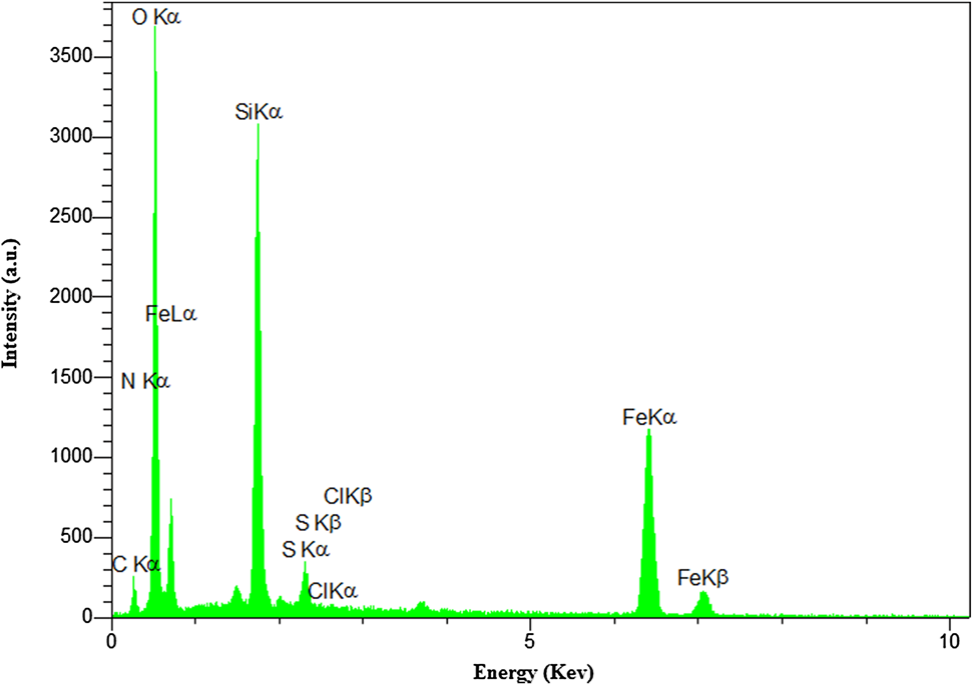
The EDS analysis of Fe_3_O_4_@Void@m.SiO_2_ nanomaterial after MB adsorption.

To investigate the successful adsorption of MB molecules into/onto Fe_3_O_4_@Void@m.SiO_2_, the EDS analysis after adsorption process was performed (Fig. 20). As shown, the existence of new peaks of carbon, nitrogen, sulfur and chlorine in this spectrum confirms successful adsorption of MB molecules into/onto material.

#### Nitrogen adsorption–desorption and BJH pore size distribution isotherms

**Figure 21:**
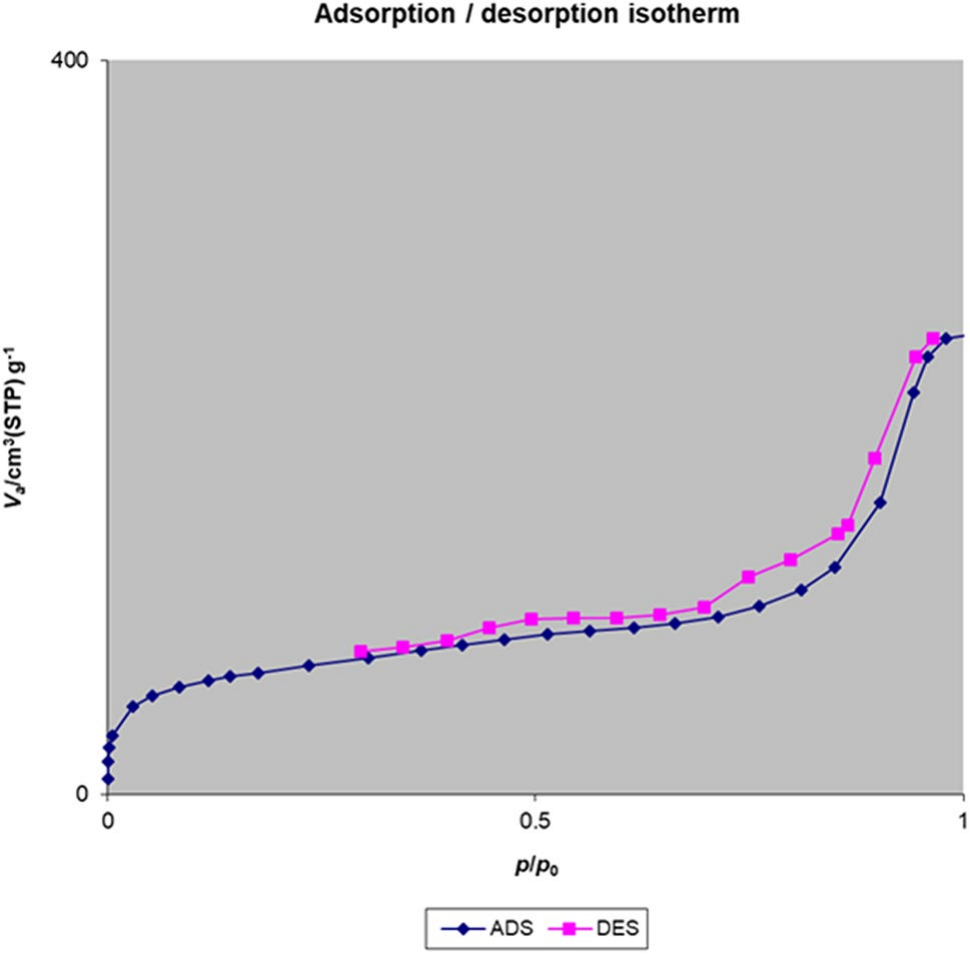
Nitrogen adsorption–desorption isotherm of the Fe_3_O_4_@Void@m.SiO_2_ nanomaterial after adsorption process.

**Figure 22:**
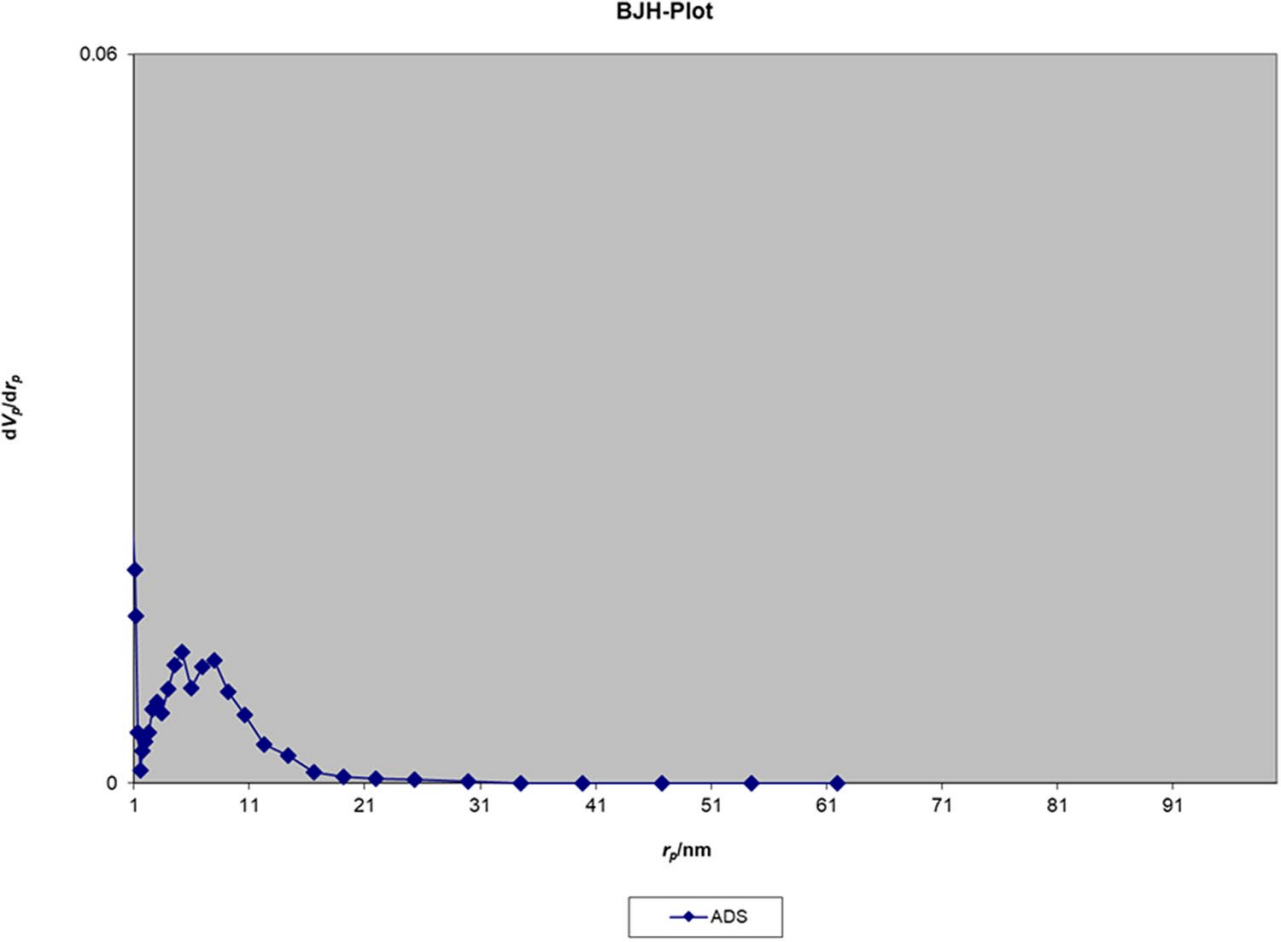
BJH pore size distribution isotherm of the Fe_3_O_4_@Void@m.SiO_2_ nanomaterial after adsorption process.

The nitrogen adsorption–desorption analysis of the Fe_3_O_4_@Void@m.SiO_2_ nanomaterial after adsorption of MB was performed (Fig. 21). According to this analysis, the BET surface area and total pore volume of the Fe_3_O_4_@Void@m.SiO_2_ nanomaterial after adsorption process were reduced to 142.32 m^2^/g and 0.35 cm^3^/g, respectively. The BJH pore size distribution isotherm after adsorption process also showed that the sizes of shell pores and void space between yolk and shell are 5.1 and 7.9, respectively (Fig. 22). Moreover, as shown in the Figs. 21 and 22, after the adsorption process the intensity of both adsorption–desorption and BJH isotherms are reduced in comparison to the fresh material. These observations confirm that both cavities and pores of the adsorbent are occupied by MB molecules.

#### Recoverability and reusability studies

**Figure 23:**
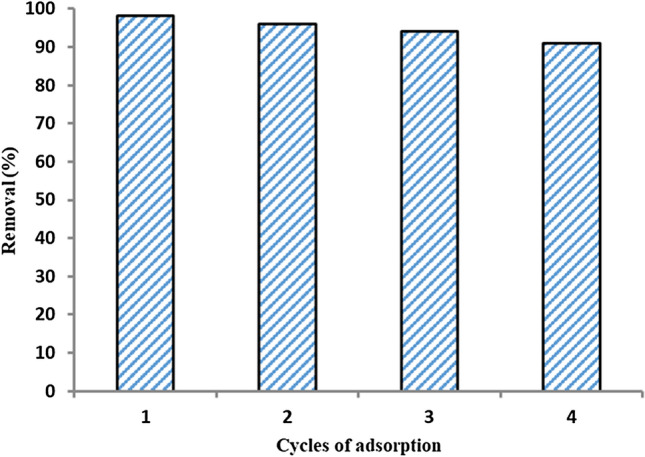
Recoverability and reusability of Fe_3_O_4_@Void@m.SiO_2_.

In next step, the recoverability and reusability of the Fe_3_O_4_@Void@m.SiO_2_ were investigated. To do this, the adsorbed MB molecules on Fe_3_O_4_@Void@m.SiO_2_ were desorbed by acidic ethanol (pH 2). Then, the adsorbent was recovered and reused under the same conditions as the first run. As Fig. 23 shown, there is an insignificant decrease in adsorption ability of adsorbent after three cycles indicating the high stability and efficiency of designed Fe_3_O_4_@Void@m.SiO_2_ for MB removal.

#### Comparison study

Next, the adsorption efficiency of our designed Fe_3_O_4_@Void@m.SiO_2_ nanomaterial was compared with some reported adsorbents in MB adsorption (Table 4). As shown, in the most of previous studies the adsorbent is not recovered and also in some cases higher temperature than RT is needed for absorption. Moreover, the time required for absorption in most of former reports is high. These findings successfully confirm higher tendency and excellent capacity of the Fe_3_O_4_@Void@m.SiO_2_ for adsorption of MB molecules in comparison to previous adsorbents.

**Table 4:** A comparison study between present adsorbent and previously reported adsorbents in the MB removal.

Entry	Adsorbent	Conditions	Adsorption efficiency (%)	Adsorption capacity (mg/g)	Recovery times	Ref
1	Graphene oxide/calcium alginate composite	3 h, 25 °C, 100 mL of 30–80 mg/L of MB solution, 0.05 g adsorbent	88.6–92.7	181.81	–	^53^
2	Activated carbon	1 h, 25 °C, 10 mL of 250–500–750 mg/L of MB solution, 0.05 g adsorbent	–	270.3	–	^54^
3	Polyaniline nanotubes base/silica composite	1 h, 25 °C, 100 mL of 3.1 mg/L of MB solution, 0.05 g adsorbent	–	0.61–5.38	–	^55^
4	Iron terephthalate (MOF-235)	1 h, 25 °C, 50 mL of 3.1 mg/L of MB solution, 0.05 g adsorbent	–	477	–	^56^
5	Nanocomposite of hydrolyzed polyacrylamide grafted xanthan gum and incorporated nanosilica	20 min, 50 °C, 25 mL of 30 mg/Lof MB solution, 0.03 g adsorbent	99.4	497.5	–	^57^
6	Fe_3_O_4_-graphene@mesoporous SiO_2_ nanocomposites	15 min, 25 °C, 25 mL of 10 mg/Lof MB solution, 0.01 g adsorbent	100	0.98–102.2	–	^58^
7	Yolk–shell tubular Fe_2_O_3_@magnesium silicate	15 h, 25 °C, 10 mL of 50 mg/L of MB solution, 0.01 g adsorbent	94	188	–	^59^
8	Fe_3_O_4_@SiO_2_ composite (adsorption–photodegradation)	5 min, 25 °C, 10 mL of 10 mg/L of MB solution, 0.01 g adsorbent	99	33.12	5	^60^
9	Yolk shell magnetic Fe_3_O_4_@hierarchical hollow silica nanomaterials	1 h, 25 °C, 50 mL of 40 mg/L of MB solution, 0.05 g adsorbent	92	71.45	5	^18^
10	Fe_3_O_4_@Void@m.SiO_2_ yolk–shell nanomaterial	15 min, 25 °C, 15 mL of 5 mg/L of MB solution, 0.005 g adsorbent	98.2	163.93	3	This work

## Conclusion

In summary, a magnetic yolk–shell structured nanomaterial with mesoporous shell (Fe_3_O_4_@Void@m.SiO_2_) was prepared, characterized and used as an effective adsorbent for the removal of MB dye from aqueous solution. The adsorption study showed that the designed material is so efficient with high removal capacity. Optimum conditions to achieve maximum removal of 98.2% for adsorption process were 0.005 g of adsorbent, pH 9 and 15 min for 15 mL of 5 mg/mL MB solution. The kinetic and isotherm studies of adsorption process were investigated and different models were evaluated for the equilibrium data. The results showed that the Langmuir isotherm model and pseudo-second-order kinetic are successfully fitted. The maximum adsorption capacity of the material was 163.93 mg/g. The comparison study illustrated that the present adsorbent is much more efficient than previously reported adsorbents in the removal of MB dye.
